# The effect of parental involvement intervention on quality of life and health outcomes among children and adolescents with chronic illness: a systematic review and meta-analysis

**DOI:** 10.1007/s00431-025-06590-y

**Published:** 2025-11-06

**Authors:** Manutsawin Kunthamas, Wanida Jaipaeng, Thatchkorn Klaisuban, Kewalin Pongsuwun, Suebsarn Ruksakulpiwat

**Affiliations:** 1 Department of Pediatric Nursing, Faculty of Nursing, Mahidol University, Bangkok, Thailand; 2 Department of Medical Nursing, Faculty of Nursing, Mahidol University, Bangkok, Thailand

**Keywords:** Parental involvement, Quality of life, Children, Adolescent, Chronic illness

## Abstract

The aim of this study is to synthesize evidence from randomized controlled (RCTs) and quasi-experimental studies evaluating the effects of parental involvement interventions on quality of life and health outcomes among children and adolescents with chronic illness. Two independent reviewers screened studies according to predefined inclusion criteria across six databases (PubMed/MEDLINE, Scopus, ScienceDirect, Cochrane Library, and Embase). Discrepancies were resolved through discussion and consensus. Meta-analysis was performed using random-effects models with mean differences (MDs) for continuous outcomes on the same scale and standardized mean differences (SMDs) for outcomes using different measurement tools. Heterogeneity was assessed using the *I*^2^ statistic, and subgroup analyses were conducted to explore potential sources of variation. This systematic review and meta-analysis included nine trials (seven RCTs and two quasi-experimental studies) involving children and adolescents aged 0–19 years with chronic illnesses, including epilepsy, asthma, type 1 diabetes, cystic fibrosis, and leukemia. Parental involvement interventions significantly improved quality of life (SMD = 1.014; 95% CI: 0.110–1.918; *p* = 0.028). Subgroup analyses revealed that shorter interventions (≤ 12 weeks) were most effective (SMD = 1.676), adolescents showed moderate improvements (SMD = 0.531), and nurse-led interventions demonstrated consistent benefits (SMD = 0.737). Effects varied significantly by economic setting, with the largest improvements in lower-middle-income countries. *Conclusion*: This study provides comprehensive evidence that parental involvement interventions significantly improve quality of life and health-related outcomes among children and adolescents with chronic illnesses, particularly when they are shorter in duration and nurse-led.

**Table:** 

What is known: •Parental involvement is essential in managing chronic illnesses in children and adolescents. •Family-centered interventions can improve adherence, psychological well-being, and coping skills.
What is new: •This review and meta-analysis show that structured parental involvement interventions significantly improve quality of life, especially when short-term and nurse-led. •Findings highlight the stronger impact in lower-middle-income countries, emphasizing equity in pediatric chronic care.

**Supplementary Information:**

The online version contains supplementary material available at 10.1007/s00431-025-06590-y.

## Background

Chronic illnesses, conditions lasting at least one year and requiring ongoing care (1), are rising among children by 0.24% annually, or about 130,000 new cases each year (2). Diseases such as asthma, type 1 diabetes, epilepsy, and inflammatory bowel disease affect not only physical health but also psychosocial development and family functioning (3–5). Children face constant treatment demands, while parents balance caregiving with supporting normal growth (6–8).

Family-centered care recognizes parents as primary caregivers and advocates in managing chronic illness (9, 10). Parental involvement includes treatment management, advocacy, and emotional support (11), but its optimal form varies. Although interventions often improve adherence, well-being, and competence (12–14), overly controlling involvement can reduce autonomy and adherence (15, 16). Effectiveness varies by intervention type, population, and developmental stage—young children require structured guidance, whereas adolescents benefit from autonomy-supportive approaches.

Understanding these dynamics is essential for designing age-appropriate, family-centered interventions. This review synthesizes evidence on parental involvement in pediatric chronic illness, assessing its impact on physical, psychological, behavioral, quality of life, knowledge, and clinical outcomes to identify mechanisms for effective, developmentally responsive care.

### Objective

The objective of this study is to synthesize and critically appraise evidence from randomized controlled and quasi-experimental studies on the effects of parental involvement interventions on quality of life and health outcomes among children and adolescents with chronic illness.

## Methods

### Search strategy

This systematic review and meta-analysis followed the PRISMA guidelines (17). A comprehensive search was conducted in PubMed/MEDLINE, Scopus, ScienceDirect, Cochrane Library, and Embase for studies published between 2020 and 2025 on parental involvement interventions for children and adolescents with chronic illnesses. Both randomized controlled trials and quasi-experimental studies were included to capture broader evidence relevant to nursing and behavioral research. Search terms are listed in Supplementary Data 1. All citations were managed in EndNote 2025 for organization and duplicate removal.

### Study selection

Records were screened in two stages: title–abstract review followed by full-text assessment using the PICOS framework and predefined inclusion and exclusion criteria. Two reviewers independently conducted screening, resolving disagreements through discussion or a third reviewer. Criteria ensuring methodological rigor and relevance are summarized in Table 1.

**Table 1:** Inclusion and Exclusion Criteria

**Inclusion Criteria**	**Exclusion Criteria**
Participants aged 0 - 19 years (studies including participants older than 19 years were acceptable if the sample included those aged 0–19). Randomized controlled trials (RCTs) or quasi-experimental studies examining the effects of parental involvement interventions on quality of life or health outcomes in children and adolescents with chronic illnesses. Studies published in English between 2020 and 2025. Studies conducted in any setting.	Studies that did not include the population of interest. Conference proceedings, pilot studies, abstracts, review articles, protocols, letters to the editor, points of view, dissertations, or grey literature. Studies involving participants who were at the end of life.

### Quality appraisal

Two reviewers independently assessed methodological rigor using the Joanna Briggs Institute (JBI) critical appraisal tools (18), applied according to study design (RCT or quasi-experimental). The tools evaluated internal validity, bias, confounding, and reliability. Each study was reviewed for design appropriateness, participant selection, randomization, measurement validity, and follow-up completeness. Discrepancies were resolved through discussion or a third reviewer, and final ratings informed interpretation and overall evidence strength.

### Data extraction

Data extraction followed a standardized template to ensure consistency and transparency. Detailed variables and procedures are provided in Supplementary Data 2.

### Data synthesis for systematic review

The review used the JBI convergent integrated analysis framework (19) to synthesize evidence from RCTs and quasi-experimental studies on parental involvement effects. Findings were organized into themes and subthemes, with quantitative data transformed into descriptive summaries for qualitative interpretation. Two reviewers independently coded and refined themes, resolving discrepancies by consensus to ensure credibility and rigor.

### Statistical analysis for meta-analysis

Data analysis followed two phases. First, a narrative synthesis summarized intervention types, participant characteristics, study designs, and outcomes. Next, a meta-analysis estimated pooled effects on quality of life and health outcomes. For continuous outcomes, mean differences (MDs) or standardized mean differences (SMDs; Cohen’s *d*) with 95% confidence intervals (CIs) were calculated. Heterogeneity was assessed using Cochran’s *Q* and *I*^2^, with ≥ 75% indicating high heterogeneity. Random-effects models were applied, with subgroup and meta-regression analyses exploring factors such as duration, delivery mode, and provider type. Sensitivity analysis (leave-one-out) tested robustness, and publication bias was examined via funnel plots and Egger’s test. All analyses used Stata 17 (StataCorp, 2021).

## Results

### Search results

**Fig. 1:**
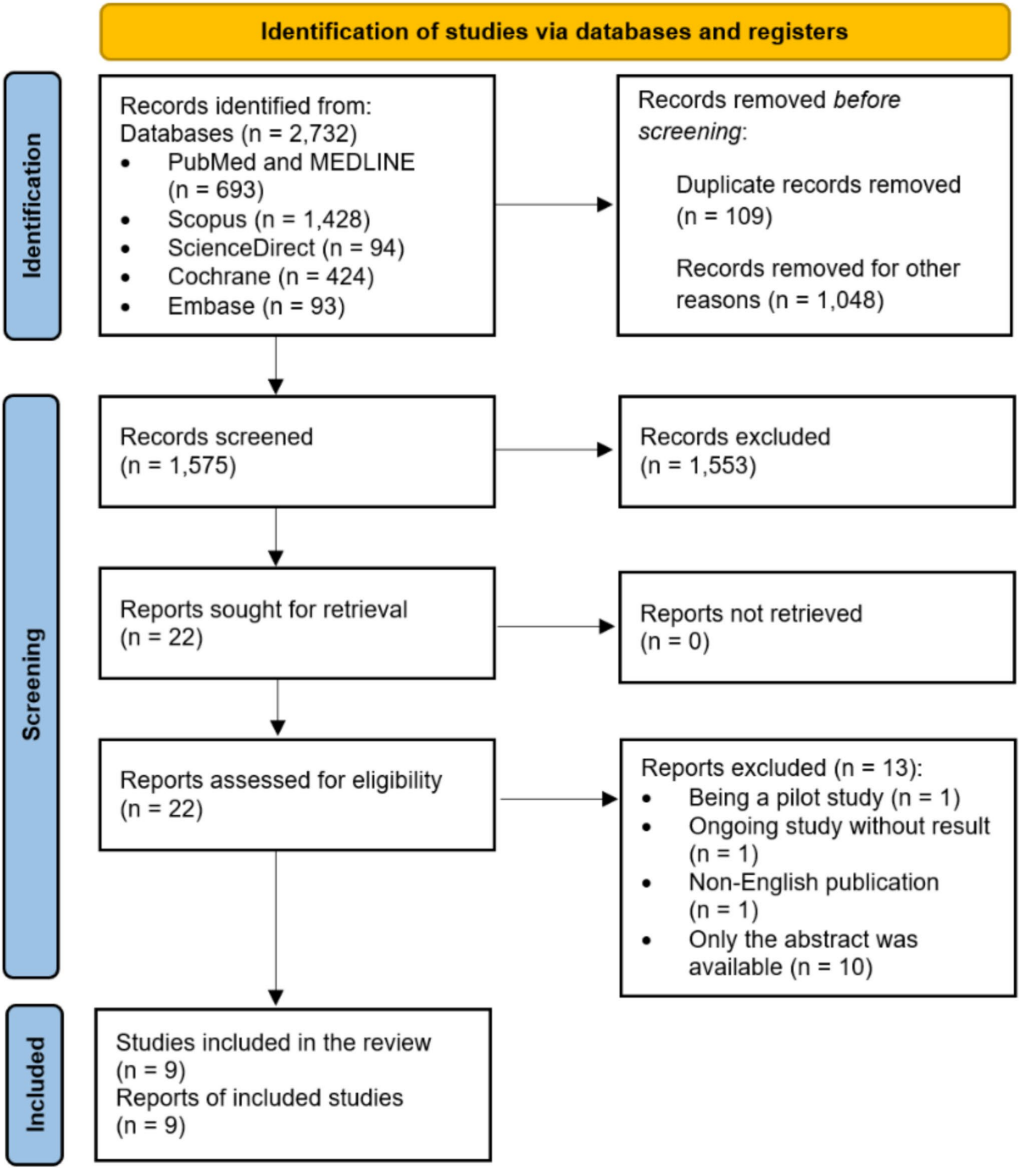
PRISMA Flow Diagram

Following PRISMA guidelines, 2,732 records were retrieved from six databases: PubMed/MEDLINE (*n* = 693), Scopus (*n* = 1,428), ScienceDirect (*n* = 94), Cochrane Library (*n* = 424), and Embase (*n* = 93). After removing 109 duplicates and 1,048 irrelevant records, 1,575 titles and abstracts were screened. Of these, 1,553 were excluded, leaving 22 articles for full-text review. Thirteen were excluded as pilot, ongoing, non-English, or abstract-only studies. Finally, nine studies met all criteria and were included. All screening was performed manually. The PRISMA flowchart (Fig. 1) summarizes the selection process.

**Adapted from**: Page MJ, McKenzie JE, Bossuyt PM, Boutron I, Hoffmann TC, Mulrow CD, et al. The PRISMA 2020 statement: an updated guideline for reporting systematic reviews. bmj. 2021;372 (20). 

### Description of included studies

Table 2 summarizes nine studies on parental involvement interventions for children and adolescents with chronic illnesses conducted across eight countries. Most were randomized controlled trials, with sample sizes ranging from 48 to 352 participants and a focus on children under 12 years. Interventions were commonly short-term (< 6 months) and nurse-led, addressing various chronic illnesses within multidisciplinary care settings

**Table 2:** Characteristic of Included Studies

**References**	**Country**	**Research Setting**	**Sample size Total, [I/C]**	**Age(Mean ± SD) [I/C]**	**% Female[I/C]**	**Study Design**	**Diagnosis**	**Provider**	**Duration (weeks)**	**Frequency**
(21)	China	Children’s Hospital	130 [65/65]	3.92 **± **3.96/3.41 **± **3.67	48.28%/42%	RCT	Epilepsy	Physicians, pharmacists, and RN	48	- Single time- F/U once a week
(22)	Iran	University Hospital	80 [40/40]	NR	NR	RCT	Epilepsy	Care providers	4	2 sessions
(23)	Germany	2 hospitals	352 [192/160]	19.8 ± 1.4/20.3 ± 2.0	49.5%/41.7%	Quasi-experimental(2-year F/U)	12 chronic illnesses	Psychologists and pediatricians	96	Single time
(15)	Netherland	3 university hospitals, 4 hospitals, and 2 schools	110 [49/71]	12.11 ± 2.73	47%	RCT	45 chronic illnesses	Psychologists	6	Once a week (90 minutes per session)
(24)	United States	2 children’s hospitals	221 [111/110]	6.4 ± 2.9/5.0 ± 2.3	37%	RCT	Asthma	Researchers	12	**1) Active Phase**:2–3 times/week**2) Reminder Phase**: once a month (9 months)
(25)	Iran	Hospital	60 [30/30]	8.46 ± 2.25/9.06 ± 2.21	43.5%/43.5%	RCT	Leukemia	Oncologists and RN	8	Once a week
26	Turkey	University hospital	48 [24/24]	10.79 ± 3.69/10.17 ± 3.5	45.8%/37.5%	RCT	Cystic Fibrosis	RN	10	1 session every 2 weeks (45–50 minutes)
(27)	Australia	Community and hospital	50 [22/28]	7.14 ± 2.12/6.39 ± 2.54	72.7%/71.4%	RCT	T1D	Psychologists and RN	24	2 sessions (2 hours)
(28)	Egypt	University children’s hospital and specialized center	328	9.85 ± 2.00	50.6%	Quasi-experimental (pre-post)	Chronic illness	Researchers and RN	12	Once a week

**Note. *** F/U* = Follow-up; *I/C* = Intervention/Control; *NR* = Not Reported; *RCT* = Randomized Controlled Trial; *RN* = Registered Nurse; *T1D* = Type 1 Diabetes.

### The quality appraisal of the included studies

The methodological quality of the included studies was assessed using the JBI critical appraisal checklist (19). Overall, the studies demonstrated moderate to high methodological quality, with an average quality rating of 72.51%. A detailed summary of the quality appraisal for each study is presented in Supplementary Data 2.

### Parental involvement intervention model

Table 3 outlines nine parental involvement models representing family empowerment, social learning, and technology-assisted self-management approaches. Structured programs included the Family Management Style, Family Empowerment, Partnership Care Model, PEINE, and Family-Centered Empowerment Program. Others used group-based psychoeducation (e.g., ModuS-T, SOK, OK, and Healthy Living Triple P) or technology-based methods such as Text2Breathe. Despite differing delivery formats, all emphasized education, empowerment, and sustained family engagement.

**Table 3:** Summary of Parental Involvement Intervention Model

**Ref**	**Named Model/Intervention**		**Core Characteristics**
	**Named Model for Parent**	**Named Model for Children**	
(21)	Family Management Style		Family-centered intervention included early care planning, in-hospital education, home self-management, and weekly follow-up.
(22)	Family Empowerment Program		Parent training covered education on disease and symptoms, seizure care, treatment, recovery, and follow-up; QoL was reassessed after one month.
(23)	ModuS-T education program		Group education with youth and parent modules using interactive methods, delivered through workshops with long-term follow-up.
(15)	"Samen Op Koers" (SOK)	"Op Koers" (OK)	**OK:** Six weekly 90-minute CBT sessions teaching age-appropriate coping skills to children, plus a 6-month booster. **SOK:** OK, plus parallel parent sessions on support, sensitivity, and motivation.
(24)	Text2Breathe		Brief in-person education using “3 Ss” communication, followed by 3 months of interactive asthma texts and 1 year of monthly vaccination and follow-up reminders.
(25)	The Partnership Care Model (PCM)		Intervention based on PCM’s four stages: family engagement, goal setting, coordinated care, and evaluation.
(26)	Parent Empowerment Intervention based on the Nursing Education (PEINE) program		Five biweekly face-to-face sessions with Q&A, weekly support calls, and an educational booklet on cystic fibrosis care for parents.
(27)	Healthy Living Triple P		Group program to enhance parenting and cooperation in T1D using behavior strategies and role-play.
(28)	Family-Centered Empowerment Program (FCEP)		Three educational sessions focused on fatigue management, family support, coping, and resilience.

**Note**. CBT = Cognitive Behavioral Therapy; QoL = Quality of life; Q&A = Question and answer; T1D = Type 1 diabetes.

## The effect of parental involvement intervention on quality of life and health outcomes (Table 4)

**Table 4:** The Summary of the Effect of Parental Involvement Interventions

**References**	**The Effect of Parental Involvement Interventions (Themes)**											
	**Primary Outcomes**						Secondary Outcomes					
	**Physiological outcome** (e.g., fatigue, strength)	**Psychological outcome** (e.g., internalizing problem, parenting stress)	**Behavioral outcome** (e.g., child’s coping skills, parents’ coping skills, competence, parenting behaviors, child behaviors)	**Quality of life** (e.g., child health-related quality of life, family quality of life)	**Knowledge** (e.g., child’s knowledge, parents’ knowledge)	**Healthcare utilization** (e.g., emergency department visit)	**Physiological outcome(e.g., metabolic control, asthma morbidity)**	**Psychological outcome** (e.g., child self-efficacy, parental self-efficacy, parenting stress)	**Behavioral outcome** (e.g., parent-child interaction, patient activation, child’s coping skills, parental coping skills, parental responsibility, child behavior)	**Quality of life** (e.g., child health-related quality of life,family quality of life)	**Healthcare utilization** (e.g., hospitalization, emergency department visit, communication with providers)	**Program Satisfaction**
**(21)**				X (Family)								
**(22)**				X (Child)								
**(23)**			X		X				X	X (Child)	X	
**(15)**		X						X	X			
**(24)**						X	X	X	X		X	
**(25)**				X (Child)								
**(26)**		X	X	X (Child)	X							
**(27)**			X	X (Child)			X	X	X	X (Family)		X
**(28)**	X							X	X	X (Child)		
**Total (%)**	1 (11.11%)	2 (22.22%)	3 (33.33%)	5 (55.56%)	2 (22.22%)	1 (11.11%)	2 (22.22%)	4 (44.44%)	5 (55.56%)	3 (33.33%)	2 (22.22%)	1 (11.11%)

### Physiological outcome (e.g., fatigue and strength)

Elbilgahy et al. evaluated a family-centered program to reduce fatigue in children with chronic illnesses and their parents (28). The intervention, based on literature-informed assessment and planning, included children with type 1 diabetes (27.4%) and cancer post-chemotherapy (24.4%). Pediatric quality of life fatigue scores rose markedly from 6.59 pre-intervention to 33.64 after three months. All fatigue subscales, general, sleep/rest, and total, showed significant improvement (*p* ≤ 0.001).

### Psychological outcome (e.g., internalizing problem and parenting stress)

Two studies (15, 26) showed mixed effects of parental involvement on psychological outcomes. Willemen et al. found that engaging parents through observation, understanding, and motivation significantly reduced children’s internalizing problems (*p* < 0.01) and parenting stress (*p* < 0.05) (15). Conversely, Donmez and Arslan reported no significant change in parenting stress after the PEINE intervention focused on education and psychological support (95% CI: − 0.221 to 0.972, *p* > 0.05) (26).

### Behavioral outcome (e.g., child’s coping skills, parents’ coping skills, competence, parenting behaviors, and child behaviors)

Three studies (23, 26, 27) examined behavioral outcomes. Mitchell et al. reported that enhancing parenting skills, confidence, and cooperation improved behaviors—reducing corporal punishment (*B* =  − 0.33, *p* < 0.05), increasing parental involvement (*B* = 0.58, *p* < 0.05), lowering child behavior problem intensity (*B* =  − 1.50, *p* = 0.014), and raising parental confidence (*B* = 8.02, *p* < 0.001) (27). Conversely, Donmez and Arslan found no significant change in parents’ coping skills (95% CI: − 0.356 to 0.831, *p* > 0.05) (26).

### Quality of life (e.g., child health-related quality of life and family quality of life)

Four studies (21, 22, 25, 26) reported quality-of-life improvements following parental involvement interventions. Donmez and Arslan found the PEINE program significantly enhanced the quality of life in children with cystic fibrosis (*p* < 0.001) (26), while Mofidi et al. using the Partnership Care Model (PCM) showed similar improvement in children with leukemia (*p* < 0.001) (25). In contrast, Mitchell et al. observed a positive trend but no significant change after the Healthy Living Triple P program (27).

### Knowledge (e.g., child’s knowledge and parents’ knowledge)

Two studies (23, 26) showed that parental involvement interventions improved illness-related knowledge in both adolescents and parents. Ernst et al. reported that the ModuS-T program significantly enhanced transition knowledge among adolescents and young adults (*F* = 18.7, *p* < 0.001) (23). Similarly, Donmez and Arslan found that parents in the PEINE program had significantly higher disease knowledge than controls (*p* < 0.001) (26).

### Healthcare utilization (e.g., emergency department visit)

Coker et al. evaluated a parent-focused text message intervention for children with chronic illness (24). At 12 months, no significant difference was found in emergency department visits between groups, but the intervention group had higher asthma-related primary care visits (95% CI: 1.03–1.76, *p* = 0.03).

## Analytical findings of meta-analysis

### Effect of parental involvement interventions on quality of life

**Fig. 2:**
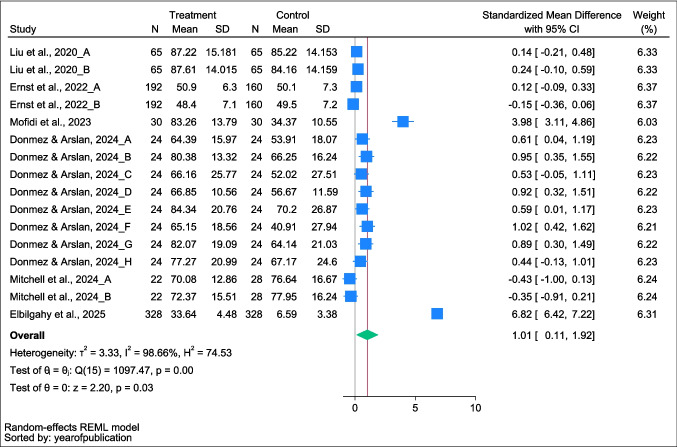
Forest plot showing the mean difference in quality-of-life scores between the parental involvement intervention group and the control group among children and adolescents with chronic illness

**Note.** The study by Donmez and Arslan (2024) reported multiple outcome measures using different subscales of quality of life, including physical functioning, emotional functioning, eating disturbances, social functioning, body image, treatment burden, respiratory symptoms, and digestive symptoms. Accordingly, each subscale was analyzed as a separate pairwise comparison (Donmez & Arslan, 2024_A to 2024_H) in the meta-analysis to capture the distinct effects of the intervention across these domains. N = number of participants; SD = standard deviation.

The meta-analysis included 16 comparisons from six studies with 1,447 participants (738 intervention, 709 control). The pooled standardized mean difference (SMD) was 1.014 (95% CI: 0.110–1.918; *p* = 0.028), showing significant improvement in quality of life among children and adolescents receiving parental involvement interventions versus standard care. Substantial heterogeneity was observed (*I*^2^ = 98.66%, The aim of this study is to = 3.33), likely influenced by intervention duration, age, provider type, and country context (Fig. 2).

### Subgroup analysis by duration of intervention

Subgroup analysis examined whether intervention duration affected outcomes. The greatest effect was observed in interventions ≤ 12 weeks (SMD = 1.676), showing significant improvement versus standard care. Longer programs of 13–48 weeks (SMD =  − 0.034) and > 48 weeks (SMD =  − 0.018) were not significant. Heterogeneity was highest for ≤ 12-week programs (*I*^2^ = 98.04%, *τ*^2^ = 4.290), largely driven by Elbilgahy et al. [28] (SMD = 6.817) and Mofidi et al. [25] (SMD = 3.982). Moderate heterogeneity appeared in 13–48 weeks (*I*^2^ = 53.72%) and higher in > 48 weeks (*I*^2^ = 68.97%). Duration differences were significant (*χ*^2^(2) = 6.44, *p* = 0.040), indicating intervention length as a potential heterogeneity source, though factors such as study design and participant traits may also contribute (Supplementary Fig. 1).

### Subgroup analysis by age group

Subgroup analysis assessed age-related differences in intervention effects. Adolescents showed a moderate, significant improvement (SMD = 0.531), while children showed a larger but nonsignificant effect (SMD = 1.729) with wide variability. Heterogeneity was substantial in both groups—higher among children (*I*^2^ = 99.40%, *τ*^2^ = 8.885) than adolescents (*I*^2^ = 74.96%, *τ*^2^ = 0.133)—driven by Elbilgahy et al. [28] and Mofidi et al. [25]. The subgroup difference was not significant (*χ*^2^(1) = 0.95, *p* = 0.330), indicating age did not moderate outcomes, though high heterogeneity warrants further sensitivity testing (Supplementary Fig. 2).

### Subgroup analysis by country

Subgroup analysis examined country income level as a moderator. The largest effect appeared in lower-middle-income countries (SMD = 5.427), followed by a moderate effect in upper-middle-income countries (SMD = 0.576), while high-income countries showed no effect (SMD =  − 0.118). Heterogeneity was highest in lower-middle-income studies (*I*^2^ = 97.01%, *τ*^2^ = 3.897), mainly influenced by Elbilgahy et al. [28] and Mofidi et al. [25]. Moderate heterogeneity was observed in high-income (*I*^2^ = 54.00%) and lower in upper-middle-income groups (*I*^2^ = 43.44%). Differences were significant (*χ*^2^(2) = 30.86, *p* < 0.001), confirming income level as a major source of heterogeneity (Supplementary Fig. 3).

### Subgroup analysis by provider

Subgroup analysis assessed provider type as a moderator. Interventions delivered by nurses with other professionals showed the largest but imprecise effect (SMD = 1.729). Nurse-only interventions demonstrated a moderate, significant effect (SMD = 0.737), while those by other professionals showed no effect (SMD =  − 0.018). Heterogeneity was absent in nurse-only studies (*I*^2^ = 0%) but extremely high in nurse–multidisciplinary programs (*I*^2^ = 99.40%, *τ*^2^ = 8.885), mainly influenced by Elbilgahy et al. [28] and Mofidi et al. [25]. Subgroup differences were significant (*χ*^2^(2) = 20.31, *p* < 0.001), confirming provider type as a major heterogeneity source (Supplementary Fig. 4).

### Sensitivity analysis

A leave-one-out analysis tested result robustness. The pooled standardized mean difference (SMD) remained consistent across iterations (0.597; 95% CI: 0.117–1.077 to 1.111; 95% CI: 0.166–2.056). Removing Elbilgahy et al. (28) caused the largest change, lowering the pooled estimate to 0.597 (95% CI: 0.117–1.077) but retaining significance (*p* = 0.015). Overall, results were stable, indicating no single study unduly influenced the findings (Supplementary Fig. 5).

### Publication bias

The funnel plot (Supplementary Fig. 6) showed slight asymmetry, with some studies having larger effects and wider standard errors, suggesting possible small-study effects. However, Egger’s test was not significant (*β* = 4.55, SE = 5.57, *z* = 0.82, *p* = 0.414), indicating no statistical evidence of publication bias. The contour-enhanced funnel plot (Supplementary Fig. 7) confirmed most effects lay in non-significant regions (*p* > 0.10), suggesting publication bias likely did not influence results.

## Discussion

### Physiological outcome

This review provides strong evidence that Family-Centered Empowerment Programs (FCEP) significantly reduce fatigue in children with chronic illnesses and their parents. Elbilgahy et al. reported major improvements across all fatigue subscales—general, sleep/rest, and cognitive (28). Similar benefits were observed in home-based multimodal programs (30), adventure-based training (31), and parental empowerment studies linking active involvement to lower pediatric fatigue (32). Collectively, parental integration into care markedly decreases fatigue and enhances recovery. Embedding family-centered empowerment in pediatric nursing practice may improve outcomes and equity. Future RCTs should confirm causality, evaluate sustainability, and test scalability across diverse healthcare systems.

### Psychological outcome

Mixed findings highlight the complexity of improving psychological outcomes through parental involvement. Donmez and Arslan reported no reduction in parenting stress (26), whereas Willemen et al. showed that active engagement reduced children’s internalizing problems and parental stress (15). These benefits likely stem from greater parental understanding, confidence, and emotional responsiveness. Similar results were reported in family empowerment programs that reduced stress and improved caregiving in thalassemia (33), asthma care using ACT-based interventions (34), and Family-Centered Empowerment Models enhancing self-efficacy and self-esteem (35). Overall, active parental engagement benefits both children and parents. Future studies should explore moderating factors (e.g., age and culture) and integrate stress-management and long-term follow-up to optimize psychological outcomes.

### Behavioral outcome

Two studies demonstrated improved behavioral outcomes from parental involvement interventions. Mitchell et al. enhanced parenting skills and cooperation, improving parenting behaviors and reducing child behavioral problems (27), while Ernst et al. addressed transition challenges among adolescents, strengthening self-management and independence (23). These findings suggest benefits across developmental stages—skill-building for younger children and autonomy support for adolescents. Supporting evidence shows that parental engagement improves self-management in asthma (34), strengthens adherence in diabetes (36), and enhances family management in thalassemia (33). Effective interventions should match developmental and family needs. Future work should integrate these models into pediatric clinics or nurse-led programs to sustain behavioral adaptation across care settings.

### Quality of life

Findings on quality of life were mixed, with several studies reporting significant improvements (21, 22, 25, 26) and one showing nonsignificant effects (27). A quasi-experimental study found that a family-management program enhanced quality of life in children with acute lymphoblastic leukemia and their families (37), consistent with similar improvements in children with autism (38) and chronic renal failure after family empowerment interventions (39). Overall, parental involvement interventions positively influence both child and family quality of life. Future studies should implement these interventions in real-world settings to assess long-term impact and sustainability.

### Knowledge

Two studies showed that parental involvement interventions improved illness-related knowledge (23, 26). These findings align with Mulyana et al., who reported that internet-based family empowerment programs enhanced parents’ understanding of chronic disease care (40). Similarly, Wang et al. found increased parental knowledge of asthma management after an ACT-based intervention (34). Lobato and Kao. also observed improved sibling knowledge and connectedness, with reduced behavioral issues in families of children with chronic illness (41). Consistently, interventions enhanced knowledge, though most outcomes were short-term. Future research should include long-term follow-up and integrate digital or mobile platforms to sustain knowledge and engagement beyond clinical settings.

### Healthcare utilization

Parental involvement interventions showed mixed effects on healthcare utilization. While they did not reduce emergency visits or asthma morbidity, they increased primary care engagement through text messaging (24). Wang et al. reported that an Acceptance and Commitment Therapy-based interventions (ACT)-based intervention improved asthma control, parental knowledge, and family empowerment (34). Similarly, the School-Based Telemedicine Enhanced Asthma Management (SB-TEAM) program reduced ED visits and improved symptoms (42), while reminder texts increased appointment adherence (43). Overall, enhanced parental understanding fosters consistent care, adherence, and fewer emergencies. Future research should identify effective components for promoting preventive care and examine long-term outcomes. Digital platforms and telehealth may further strengthen engagement and continuity of care.

## Meta-analysis findings and implications

This meta-analysis showed that parental involvement interventions significantly improved quality of life in children and adolescents with chronic illnesses (28, 44). However, substantial heterogeneity reflects differences in design, participants, and settings. Shorter interventions (≤ 12 weeks) produced the greatest benefit, while longer programs showed no significant effect, suggesting that intensive, time-limited models may be more feasible and impactful (45–47).

By age, adolescents had moderate, significant improvement, whereas children showed a larger but nonsignificant effect, highlighting developmental differences and the need for age-tailored strategies (15, 48). Context also influenced outcomes: effects were strongest in lower-middle-income countries, moderate in upper-middle-income, and nonsignificant in high-income settings (49–53), emphasizing the importance of socioeconomic context and available support systems.

Provider type further moderated outcomes. Nurse-led interventions consistently improved results, confirming their reliability and impact, while multidisciplinary and other provider-led programs were less consistent (54–56). Overall, shorter, nurse-led parental involvement programs effectively enhance pediatric quality of life and health outcomes. Future research should standardize intervention components, assess sustainability, and evaluate contextual and individual moderators to maximize global applicability.

## Conclusion

Parental engagement is essential in pediatric chronic illness management, improving adherence, care, and well-being. This review shows that structured parental involvement interventions significantly enhance quality of life. Integrating these programs into routine care strengthens family-centered practice, supports equity, and provides lasting benefits for children and adolescents with chronic illness.

### Limitations

This review has several limitations. Combining randomized and quasi-experimental studies introduced methodological heterogeneity and possible bias, including Hawthorne effects. Variations in study quality and outcome measures limited comparability, underscoring the need for rigorous randomization, blinding, and standardized tools. The five-year search window may have excluded earlier interventions, and limited follow-up restricted assessment of long-term effects. Restricting to English-language studies may have introduced language and publication bias, underrepresenting culturally diverse models.

## Supplementary Information

Below is the link to the electronic supplementary material.

ESM1 (DOCX.118 KB)

ESM2 (DOCX.17.4 KB)

ESM3 (DOCX.45.5 KB)

## Data Availability

The supplementary data for this study is available in the online version of the article and can also be requested from the corresponding author.

## Abbreviations

CIs: Confidence intervals
ED: Emergency department
FCEP: Family-Centered Empowerment Program
JBI: Joanna Briggs Institute
MDs: Mean differences
*N*: Number of participants
OK: Op Koers
PCM: The Partnership Care Model
PEINE: Parent Empowerment Intervention based on the Nursing Education program
PRISMA: Preferred Reporting Items for Systematic Reviews and Meta-Analyses guidelines
RCT: Randomized controlled trial
SB-TEAM: School-Based Telemedicine Enhanced Asthma Management program
SD: Standard deviation
SMD: Standardized mean differences
SOK: Samen Op Koers
