# Supplementary material for: The effect of parental involvement intervention on quality of life and health outcomes among children and adolescents with chronic illness: a systematic review and meta-analysis
Source: Eur J Pediatr. 2025 Nov 6;184(12):733. doi: 10.1007/s00431-025-06590-y (PMC12589278; doi:10.1007/s00431-025-06590-y)
Supplement: Supplementary file 1 — (DOCX.118 KB) [file 431_2025_6590_MOESM1_ESM.docx]

**Supplementary Data 1.** Search Term

**Keywords:** (Children OR Child OR Adolescents OR Adolescence OR Adolescents, Female OR Adolescent, Female OR Female Adolescent OR Female Adolescents OR Adolescents, Male OR Adolescent, Male OR Male Adolescent OR Male Adolescents OR Youth OR Youths OR Teens OR Teen OR Teenagers OR Teenager) AND (Chronic Diseases OR Disease, Chronic OR Chronic Illness OR Chronic Illnesses OR Illness, Chronic OR Chronically Ill OR Chronic Condition OR Chronic Conditions OR Condition, Chronic) AND (Parental involvement OR Parental engagement OR Family engagement OR Parent-involved OR Parent participation) AND (Intervention OR Experimental OR program OR method)

| **Database** | **Search strings** |
| --- | --- |
| **PubMed/MEDLINE** | (("Children"[Mesh] OR "Child"[Mesh] OR "Adolescent"[Mesh] OR Children OR Child OR Adolescents OR Adolescence OR Youth OR Youths OR Teen OR Teens OR Teenager OR Teenagers)  AND  ("Chronic Disease"[Mesh] OR "Chronic Diseases"[Mesh] OR Chronic Illness OR Chronic Illnesses OR Chronically Ill OR Chronic Condition OR Chronic Conditions)  AND  ("Parental Involvement"[Mesh] OR "Family"[Mesh] OR Parental involvement OR Parental engagement OR Family engagement OR Parent-involved OR Parent participation)  AND  (Intervention OR Experimental OR Program OR Method)) |
| **Scopus** | (TITLE-ABS-KEY (Children OR Child OR Adolescents OR Adolescence OR Youth OR Teen OR Teenagers)  AND  TITLE-ABS-KEY ("Chronic Disease" OR "Chronic Illness" OR "Chronic Condition" OR "Chronic Diseases")  AND  TITLE-ABS-KEY ("Parental involvement" OR "Parental engagement" OR "Family engagement" OR "Parent-involved" OR "Parent participation")  AND  TITLE-ABS-KEY (Intervention OR Experimental OR Program OR Method)) |
| **ScienceDirect** | ("Children" OR "Child" OR "Adolescent" OR "Adolescents" OR "Youth" OR "Teenagers")  AND  ("Chronic Disease" OR "Chronic Illness" OR "Chronic Condition")  AND  ("Parental involvement" OR "Parental engagement" OR "Family engagement" OR "Parent-involved" OR "Parent participation")  AND  (Intervention OR Experimental OR Program OR Method) |
| **Cochrane Library** | ([mh Children] OR [mh Child] OR [mh Adolescent] OR Children OR Adolescents OR Teen OR Youth)  AND  ([mh Chronic Disease] OR Chronic Illness OR Chronic Condition)  AND  (Parental involvement OR Parental engagement OR Family engagement OR Parent-involved OR Parent participation)  AND  (Intervention OR Experimental OR Program OR Method) |
| **Embase** | ('child'/exp OR 'children' OR 'adolescent'/exp OR adolescent OR adolescence OR youth OR teen OR teenager)  AND  ('chronic disease'/exp OR 'chronic illness' OR 'chronic condition')  AND  ('parent involvement'/exp OR 'parent engagement' OR 'family engagement' OR 'parent-involved' OR 'parent participation')  AND  ('intervention' OR 'experimental' OR 'program' OR 'method') |

**Note:** The search strategies were adapted to match the indexing terms and syntax of each database.
