# Supplementary material for: The effect of parental involvement intervention on quality of life and health outcomes among children and adolescents with chronic illness: a systematic review and meta-analysis
Source: Eur J Pediatr. 2025 Nov 6;184(12):733. doi: 10.1007/s00431-025-06590-y (PMC12589278; doi:10.1007/s00431-025-06590-y)
Supplement: Supplementary file 3 — (DOCX.45.5 KB) [file 431_2025_6590_MOESM3_ESM.docx]

**Supplementary Data 2**

| **Authors, year**  **(Ref)**      **Data** | **Liu et al., 2020**  **(21)** | **Nemati et al., 2021**  **(22)** | **Ernst et al., 2022**  **(23)** | **Willemen et al., 2022**  **(15)** | **Coker et al., 2023**  **(24)** | **Mofidi et al., 2023**  **(25)** | **Donmez & Arslan, 2024**  **(26)** | **Mitchell et al., 2024**  **(27)** | **Elbilgahy et al., 2025**  **(28)** |
| --- | --- | --- | --- | --- | --- | --- | --- | --- | --- |
| **Title** | Family Management Style Improves Family Quality of Life in Children with Epilepsy: A Randomized Controlled Trial | The Effect of Family Empowerment Model on Quality of Life in Children with Epilepsy in South of Iran, 2018: A Randomized Controlled Clinical Trial | Two-Year Follow-Up of a Transition-Specific Education Program for Young People with Chronic Conditions | The Working Mechanisms of Parental Involvement in Interventions for Children with Chronic Illness | Text2Breathe: Text-Message Intervention for Parent Communication and Pediatric Asthma | Effect of a partnership care  programme on quality of life in  school-age children with leukaemia:  a controlled clinical trial | The Effectiveness of a Parent Empowerment Intervention for Caregivers of Children with Cystic Fibrosis | Randomised controlled trial of the Healthy Living Triple P-Positive Parenting Program for families of children with type 1 diabetes | Empowering families: the role of family-centered programs in alleviating fatigue for chronically ill children and their parents |
| **Country** | China | Iran | Germany | Netherlands | United States | Iran | Turkey | Australia | Egypt |
| **Setting** | The neurology department of Hunan Children's Hospital in Changsha, Hunan Province, China | The Bessat  Clinic affiliated to the Kerman University of Medical Sciences and Shiraz’s Imam Reza Clinic | 29 adult and pediatric outpatient clinics, two hospitals | Outpatient clinics of three academic hospitals, four non-academic hospitals, and two primary schools | Emergency Department of 2 children’s hospitals in Seattle and Tacoma | Paediatric ward of Shafa Hospital in  Ahvaz, Iran, | The pediatric chest diseases clinic of a university hospital | Community and hospital outpatient clinics | Mansoura University Children’s Hospital and Mansoura Oncology Center |
| **Study design** | RCT | RCT | Prospective, longitudinal controlled study with 2-year follow-up | RCT | RCT | Quasi-experimental (single group pre/post-test) | RCT | RCT | Quasi-experimental (single group pre/post-test) |
| **Diagnosis** | Epilepsy | Epilepsy | 12 different chronic conditions (i.e., asthma, ADHD, chronic kidney disease, cystic fibrosis, type 1 diabetes, Ehlers-Danlos syndrome, epilepsy, esophageal atresia, inflammatory bowel disease, juvenile idiopathic arthritis, liver/kidney transplantation, phenylketouria) | 45 different chronic illnesses. The three most common ones were: type 1 diabetes, autoimmune diseases, and kidney disease | Asthma | Leukemia | Cystic fibrosis | Type 1 diabetes | Chronic illnesses (e.g., type 1 diabetes, cancer, etc.) |
| **Age [I/C] (M±SD)** | 3.92 **±** 3.96/  3.41 **±** 3.67 | Not Reported | 19.8 ± 1.4/  20.3 ± 2.0 | 12.11 ± 2.73 | 6.4 ± 2.9/    5.0 ± 2.3 | 8.46 ± 2.25/  9.06 ± 2.21 | 10.79 ± 3.69/  10.17 ± 3.5 | 7.14 ± 2.12/  6.39 ± 2.54 | 9.85 ± 2.00 |
| **Female**  **[I/C] (%)** | 48.28% / 42% | Not Reported | 49.5% / 41.7% | 47% | 37% | 43.5% / 43.5% | 45.8% / 37.5% | 72.7% / 71.4% | 50.6% |
| **Sample size allocation [I/C]** | 65/65 | 40/40 | 192/160 | 49/71 | 111/110 | 30/30 | 24/24 | 22/28 | 328 |
| **Purpose** | To analyze the effectiveness of family management style on family quality of life in children with epilepsy. | To evaluate the effect  of family empowerment on the quality of life of epileptic children referred to the concerned centers. | To assess long-term effects (after 2 years) of the ModuS-T education program on transition-specific knowledge, competencies, patient activation, and continuity of medical care in young people with chronic conditions. | To determine whether active coping skills and stress in the parent-domain mediated the effect of the parent–child intervention (SOK) on child internalizing problems over child-only intervention (OK). | To test the impact of a text messaging program on emergency department utilization and asthma morbidity. | To evaluate the effect of a care  program based on the PCM on the QoL of children with leukemia | to evaluate the effects of a nursing education-based empowerment program applied to the parents of children with CF on problem-solving and stress coping in parents, as well as on health-related quality of life in children with CF. | To evaluate the efficacy of the Healthy Living Triple P – Positive Parenting Program in improving parenting practices, child adjustment, family functioning, and health-related outcomes among families of children with type 1 diabetes. | To assess the impact of a family-centered empowerment program (FCEP) on reducing fatigue levels in children with chronic illnesses and their parents. |
| **Intervention model/name** | Family Management Style | Family Empowerment Program | ModuS-T education program | "Samen Op Koers" (SOK) for parent and "Op Koers" (OK) for children | Text2Breathe | The Partnership Care Model (PCM) | Parent empowerment intervention based on the nursing education (PEINE) program | Healthy Living Triple P | Family-Centered Empowerment Program (FCEP) |
| **Intervention provider(s)** | Physicians, pharmacists, and nurses | Care providers | Psychologists and pediatricians | Psychologists | Researchers | Oncologists and nurses | Nurses | Psychologists and nurses | Researchers and nurses |
| **Intervention duration** | 12 months  (48 weeks) | 1 month  (4 weeks) | 24 months  (96 weeks) | 6 weeks | 12 months  (48 weeks) | 2 months  (8 weeks) | 10 weeks | 24 weeks | 3 months  (12 weeks) |
| **Intervention detail(s)** | Family-centered intervention included early care planning, in-hospital education, home self-management, and weekly follow-up. | Parent training covered education on disease and symptoms, seizure care, treatment, recovery, and follow-up; QoL was reassessed after one month. | Group education with youth and parent modules using interactive methods, delivered through workshops with long-term follow-up. | **OK:** Six weekly 90-minute CBT sessions teaching age-appropriate coping skills to children, plus a 6-month booster.  **SOK:** OK, plus parallel parent sessions on support, sensitivity, and motivation. | Brief in-person education using “3 Ss” communication, followed by 3 months of interactive asthma texts and 1 year of monthly vaccination and follow-up reminders. | Intervention based on PCM’s four stages: family engagement, goal setting, coordinated care, and evaluation. | Five biweekly face-to-face sessions with Q&A, weekly support calls, and an educational booklet on cystic fibrosis care for parents. | Group program to enhance parenting and cooperation in T1D using behavior strategies and role-play. | Three educational sessions focused on fatigue management, family support, coping, and resilience. |
| **Intervention frequency** | - Single time  - Follow-up once a week | 2 sessions | Single time | Once a week (90 minutes per session) | 1) Active Phase:  2–3 times/week  2) Reminder Phase: once a month (9 months) | Once a week | 1 session every 2 weeks   (45-50 minutes) | 2 sessions   (2 hours each over 1 week) | Once a week |
| **Intervention of control group** | Routine Care as conventional disease management and monthly telephone follow-ups | The educational pamphlet was submitted to the parents to observe the ethical issues. | Control group received usual care, including individual medical care and counselling. | No control group | usual care with 12 months of text message visit reminders | Usual care | The control group received routine care. Routine care involves regular respiratory function tests, lung radiography, blood tests, and treatment planning. | Care-as-usual (waitlist control) | No control group |

**Primary and Secondary Measurements**

| **Authors, year**  **(Ref)**  **Data** | | **Liu et al., 2020**  **(21)** | **Nemati et al., 2021**  **(22)** | **Ernst et al., 2022**  **(23)** | **Willemen et al., 2022**  **(15)** | **Coker et al., 2023**  **(24)** | **Mofidi et al., 2023**  **(25)** | **Donmez & Arslan, 2024**  **(26)** | **Mitchell et al., 2024**  **(27)** |
| --- | --- | --- | --- | --- | --- | --- | --- | --- | --- |
| **Primary measurement(s)** | **Physiological outcome** |  |  |  |  |  |  |  |  |
|  | **Psychological outcome** |  |  |  | 1. Child behavior checklist youth self-report (CBCL-YSR) problems  2. Child behavior checklist parent report form (CBCL-PRF) |  |  |  |  |
|  | **Behavioral outcome** |  |  | Transition Competence Scale (TCS) |  |  |  | Coping Skills (WCI scores) | 1. Parenting behavior (APQ)  2. child behavior/  adjustment (CAPES) |
|  | **Pediatric quality of life (QoL)** |  | The Quality of Life in  Childhood Epilepsy Questionnaire (QOLCE) |  |  |  | PedSQL | Quality of Life in Children (CFQ-R scores) | Child HRQoL (PedsQL). |
|  | **Others** | The Beach Center Family Quality of Life Scale (FQOL Scale) (Chinese version) |  | Transition Knowledge Test |  | Parents' report |  | 1. Parenting Stress (PSI scores)  2.Coping Skills (WCI scores) |  |
| **Secondary measurement(s)**  **Secondary measurement(s)** | |  |  | 1. Patient Activation Measure – Short Form (PAM-13)  2. German standard version of the Short Form 8 (SF-8)  3. Self-reported questionnaires  4. Self-developed questionnaire | 1. Questionnaire Op Koers for children (QOK-c)  2. Parenting stress index short form (PSI-SF) | 1. The Inner-City Asthma Consortium validated Questionnaire  2. The Medical Competence Communication Scale  3. Parent’s Report  4. The Asthma Self-Management Knowledge Questionnaire (ASMQ) |  | 1. Metabolic control (BGL, HbA1c)  2. diabetes self-efficacy (SED)  3. T1D-specific child behavior (DBC)  4. family QoL (PedsQL-FIM)  5. parenting stress (PSI)  6. parent-child interactions (MOS/ECES)  7. program satisfaction. | 1. Child quality of life, parental coping and self-efficacy: Checklist Individual Strength (CIS) subscales  2. Observational indicators |

**Primary and Secondary Results**

| **Authors, year**  **(Ref)**  **Data** | | **Liu et al., 2020**  **(21)** | **Nemati et al., 2021**  **(22)** | **Ernst et al., 2022**  **(23)** | **Willemen et al., 2022**  **(15)** | **Coker et al., 2023**  **(24)** | **Mofidi et al., 2023**  **(25)** | **Donmez & Arslan, 2024**  **(26)** | **Mitchell et al., 2024**  **(27)** | **Elbilgahy et al., 2025**  **(28)** |
| --- | --- | --- | --- | --- | --- | --- | --- | --- | --- | --- |
| **Primary result(s)** | **Physiological outcome** |  |  |  |  |  |  |  |  | Significant reduction in fatigue among children: Mean PedsQL-MFS score increased from 6.59 ± 3.38 to 33.64 ± 4.48 (p ≤ .001); significant improvements in all subscales: general, sleep/rest, and cognitive fatigue |
|  | **Psychological outcome** |  |  |  | 1. SOK intervention showed better effectiveness compared to OK intervention  2. Both child-reported and parent-reported internalizing problems decreased more significantly in the SOK intervention group |  |  |  |  |  |
| **Primary result(s)**  **Primary result(s)** | **Behavioral outcome**  **Behavioral outcome** |  |  | Intervention group showed significantly higher competencies score compared to the control group after two years of the program (F = 55.3, p < .001) |  |  |  | Coping Skills (WCI scores): No significant difference between groups after the intervention d = 0.239 [CI: −0.356, 0.831] (p > .05) | **Parenting Behavior (APQ – Parent and Child Reports):**  Reduction in corporal punishment (parent-report) in the intervention group was statistically significant:  → Group × Time interaction: B = −0.33, F(1, 83.19) = 4.00, p = .049  → Post hoc (intervention): B = −0.43, p = .005; Control group: B = −0.11, p = .269  **Improvement in parental involvement over time (regardless of group):**  → B = 0.58, F(1, 41.15) = 4.60, p = .038  **Child-report of corporal punishment showed significant decrease over time:**  → B = −0.60, F(1, 66.18) = 6.72, p = .012 (no significant group × time effect)  **Child Behavior and Parental Confidence (CAPES – Parent Report)**  **Reduction in behavior problem intensity over time:**  → B = −1.50, F(1, 43.25) = 6.49, p = .014  **Increase in parental confidence over time:**  → B = 8.02, F(1, 38.61) = 16.13, p < .001 |  |
| **Primary result(s)**                                              **Primary result(s)** | **Pediatric quality of life (QoL)** |  | Data in Table was not clearly. "These results revealed a statistically significant difference between the two groups regarding the level of changes in all dimensions, indicating that the experimental group’s mean score of quality of life in different dimensions increased (p < .05 in all dimensions)." |  |  |  | The total QoL score and subscale scores significantly improved from pre-test to post-test in the  experimental group (p < .001) | Children in the intervention group had significantly higher quality of life scores    d = 1.363 [CI: 1.698, 2.015] (p < .001) | **Child Quality of Life (PedsQL – Child Report):**  No statistically significant changes over time or between groups. |  |
|  | **Others**                      **Others** | Full scores and each subscale's scores on FQOL in the control group and the intervention group at T1 had no statistical significance (p > .05). Scores on FQOL at T2 and T3 increased in the intervention group, but there was almost no change in the control group, with statistical significance between the intervention group and the control group (p < .05). Scores on FQOL at T1, T2, and T3 showed that score of subscales except parenting FQOL improved in the intervention group (p < .05), but no difference was shown in the control group (p > .05). There was no difference shown among the control group and the intervention group that interacted with time (p > .05) |  | Intervention group showed a significant improvement in transition-specific knowledge score compared to the control group after two years of the program (F = 18.7, p < .001). |  | Number of Emergency Department (ED) Visits IRR: 1.19 (95% CI: 0.86 to 1.63) (p = .29) |  |  |  |  |
| **Secondary result(s)**                                                **Secondary result(s)** | |  |  | After two years of the program  1. Patient activation increased significantly in the intervention group (F = 14.0, p < .001)  2. No significant difference of health-related quality of life between intervention and control group (p > .05)  3. Not all findings (number of physician visits, missed medical appointments, emergency care usage, hospitalizations, and continuity of care after transition) were statistically significant, but the intervention group tended to remain more engaged with care services, especially adult-care providers  4. Results showed a significant reduction in parental disease responsibility in the intervention group (F = 8.3, p < .01) | 1. Active coping skills    - social competence and information seeking significantly mediated intervention effects on both child- and parent-reported internalizing problem   - children in the SOK group showed significant increases in social competence and information seeking as compared to the OK group  - no significant indirect effects were found of positive thinking on both child- or parent-reported internalizing problems  2. Parenting stress   - parenting stress significantly mediated intervention effects on child- and parent-reported internalizing problem  - parents in the SOK group showed a significantly stronger decrease in parenting stress as compared to the OK group | 1) Morbidity (Maximum Symptom Days) IRR: 1.32 (95% CI: 0.89 to 1.97) (p = .16)  2) Number of Visits with Primary Care Provider (PCP) for Preventive Asthma Care in the Past 12 Months IRR: 1.35 (95% CI: 1.03 to 1.76) (p = .03)  3) Parental Communication Self-Efficacy Score Mean Difference: –0.09 (95% CI: –0.96 to 0.77) (p = .83)  4) Parental Asthma Self-Management Knowledge Mean Difference: –0.01 (95% CI: –4.82 to 4.80) (p = .99) |  |  | The program showed limited effects on clinical outcomes (e.g., HbA1c) and other behavioral indicators. High program acceptability was reported, though measured qualitatively. | Significant improvement in child QoL and parental coping/self-efficacy (p < .05); no significant change in stress |

**Advantages, Implication, Limitations, and Quality appraisal**

| **Authors, year**  **(Ref)**    **Data** | **Liu et al., 2020**  **(21)** | **Nemati et al., 2021**  **(22)** | **Ernst et al., 2022**  **(23)** | **Willemen et al., 2022**  **(15)** | **Coker et al., 2023**  **(24)** | **Mofidi et al., 2023**  **(25)** | **Donmez & Arslan, 2024**  **(26)** | **Mitchell et al., 2024**  **(27)** | **Elbilgahy et al., 2025**  **(28)** |
| --- | --- | --- | --- | --- | --- | --- | --- | --- | --- |
| **Advantages** | 1. Best design for interventional study.  2. The intervention could improve FQOL, emotional well-being and support. | The implementation of the family-centered care plan by care providers, based on patient-family support relationships, the detection of their strengths and weaknesses, prioritization of the provided services, and effective interaction with the health team would increase the family and staff’s satisfaction, reduce the costs, and improve the outcome of the disease. | 1. Sustained improvement of transition-specific competency and knowledge  2. Increased activation and engagement in healthcare services  3. Perceived responsibility  : a reduction of parental involvement over time would reflect increased autonomy of the young person. | 1. Involving parents in teaching and reinforcing coping strategies, while simultaneously addressing parental stress, can improve psychological outcomes for children with chronic illness  2. Enhancing parental well-being | 1. Demonstrated Impact on Preventive Care Use: highlighting its potential to improve preventive health behavior.  2. Longitudinal Follow-Up: 12 months, allowing researchers to observe sustained behavior changes and outcomes over time. | 1. Well-controlled design  2. All participants were treated at the same medical center, reducing variability in care settings. | 1. Focused on Empowerment through Education  2. Improved Disease Knowledge  3. Enhanced Quality of Life in Children | The use of an evidence-based parenting intervention (Triple P) tailored specifically for families managing type 1 diabetes represented a key advantage, as did the inclusion of both primary and secondary caregivers, which allowed for a broader understanding of family dynamics. The intervention was also well-received by participants, indicating good feasibility and acceptability. | The family-centered empowerment program was structured, interactive, and culturally tailored to children and parents. It enhanced knowledge, coping, and self-efficacy using group-based sessions and validated tools (PedsQL-MFS, CIS). Its delivery in clinical settings and emphasis on collaborative care make it feasible, engaging, and scalable across similar healthcare environments. |
| **Future research/**  **clinical implication**  **Future research/**  **clinical implication** | Broader studies are needed to confirm the intervention’s effectiveness and feasibility. | Patient and family empowerment has been of great significance in nursing and medical studies as such it should be considered as an essential part of the nursing profession. | 1. Encourages integration of such programs into routine care for adolescents with chronic conditions.  2. Recommends further scaling and institutionalization of ModuS-T as part of structured transition care pathways in order to reduce gaps in care during transition from pediatric to adult services.  3. Emphasizes the importance of adapting educational content to the specific needs and conditions of young people. | 1. Parental involvement enhances treatment effectiveness through specific mechanisms rather than general support.  2. Parent-child interventions should be prioritized as a more beneficial treatment option compared to child-only intervention, especially, focusing on improving child active coping skills and reducing parenting stress  3. Parent involvement facilitates real-world application and maintenance of coping skills beyond therapy completion. | 1. Need for Multifaceted Approaches  2. Customization and Technological Adaptability: Such as interpreting user responses or providing real-time feedback, to better personalize content and increase engagement.  3. Inclusion of Additional Outcome Measures: should include broader outcomes such as asthma-related quality of life, school attendance, and caregiver burden, to better capture the holistic impact of interventions. | 1. Need to evaluate the effects of similar PCM-based programs on QoL in children with leukemia with a larger sample size, longer follow-up periods and an active control group | 1. Future research should explore longer-term impacts, include psychological interventions, and consider digital delivery methods. Clinically, PEINE demonstrates the important role nurses can play in educating and empowering families, improving outcomes for children with CF. | 1. Future studies should assess longer-term outcomes and focus on clinical indicators such as glycemic control. Additional adaptation and evaluation of the intervention across diverse populations and caregiver roles are also warranted. | Future research should use randomized controlled trials to validate findings and assess long-term outcomes. Clinically, integrating such programs into routine care can improve treatment adherence, reduce caregiver fatigue, and enhance child quality of life. Training healthcare professionals in family empowerment strategies is recommended to promote sustainable, family-centered chronic care models. |
| **Limitation**  **Limitation** | 1. The family management style relied on parents to apply it at home, so researchers couldn’t continuously monitor the children’s behaviors and skills outside the hospital.        2. The study used self-reported questionnaires, which may lead to biased responses due to exaggeration or reluctance to share private information.      3. It was a single-center study with participants only from one hospital, which could cause selection bias. | 1. Double blind RCT was not applied.  2. A small sample size could not represent the generalizability of the population. | 1. The study was non-randomized design, which could introduce selection bias.  2. Attrition and follow-up rates: there was moderate drop-out over the 2-year follow-up period, which may affect the generalizability of long-term findings.  3. The study utilized many self-reports questionnaires, which may be subject to social desirability or recall bias. | 1. Lacking of objective measure: child-reported coping skills represented the subjective perception of the use of the skills by children  2. Small to medium effect sizes suggest other unmeasured mechanisms may be important  3. Potential selection bias due to some randomized groups not being conducted  4. Brief intervention duration may limit ability to change certain internal processes | 1. Slow Recruitment Timeline  2. Technological Limitations  3. Baseline Age Imbalance  4. Hawthorne Effect: except for PCP utilization.  5. Recall Bias  6. Unmeasured Outcomes: Important outcomes such as asthma-related quality of life were not assessed in this study. | 1. Lack of double-blinding  2. Small sample size  3. Attention bias | The study’s small sample size and short duration may limit how broadly the results apply and the ability to assess long-term effects. It relied on self-reported data, which can introduce bias. Children were not included in the intervention sessions, potentially overlooking family dynamics. Finally, conducting the study in a single clinic limits generalizability to other settings or populations. | The small sample size and brief duration of the intervention limit the generalizability of the findings. Moreover, no significant improvements were observed in metabolic outcomes such as HbA1c. The use of self-reported measures and lack of blinding may have introduced response bias, which should be addressed in future trials. | This quasi-experimental study lacked randomization and a control group, limiting causal inference. Self-reported measures may introduce bias, and the six-month follow-up was short for evaluating sustainability. Conducted at a single site, generalizability is limited. Fathers and other caregivers were excluded, reducing the comprehensiveness of the family-based outcome evaluation. |
| **Quality appraisal by**  **JBI (%)** | 76.92% | 46.15% | 88.89% | 69.23% | 66.15% | 89% | 69.23% | 69.23% | 77.80% |
